# A Data Matrix Code Recognition Method Based on L-Shaped Dashed Edge Localization Using Central Prior

**DOI:** 10.3390/s24134042

**Published:** 2024-06-21

**Authors:** Yi Liu, Yang Song, Guiqiang Gu, Jianan Luo, Taoan Wang, Qiuping Jiang

**Affiliations:** 1 College of Science and Technology, Ningbo University, Ningbo 315300, China; liuyi@nbu.edu.cn (Y.L.); songyang@nbu.edu.cn (Y.S.); ggq2652104553@163.com (G.G.); l228986872@gmail.com (J.L.); taoan0315@163.com (T.W.); 2 School of Information Science and Engineering, Ningbo University, Ningbo 315211, China

**Keywords:** data matrix code, localization, recognition, industrial production, timing pattern, L-shaped solid and dashed edges

## Abstract

The recognition of data matrix (DM) codes plays a crucial role in industrial production. Significant progress has been made with existing methods. However, for low-quality images with protrusions and interruptions on the L-shaped solid edge (finder pattern) and the dashed edge (timing pattern) of DM codes in industrial production environments, the recognition accuracy rate of existing methods sharply declines due to a lack of consideration for these interference issues. Therefore, ensuring recognition accuracy in the presence of these interference issues is a highly challenging task. To address such interference issues, unlike most existing methods focused on locating the L-shaped solid edge for DM code recognition, we in this paper propose a novel DM code recognition method based on locating the L-shaped dashed edge by incorporating the prior information of the center of the DM code. Specifically, we first use a deep learning-based object detection method to obtain the center of the DM code. Next, to enhance the accuracy of L-shaped dashed edge localization, we design a two-level screening strategy that combines the general constraints and central constraints. The central constraints fully exploit the prior information of the center of the DM code. Finally, we employ libdmtx to decode the content from the precise position image of the DM code. The image is generated by using the L-shaped dashed edge. Experimental results on various types of DM code datasets demonstrate that the proposed method outperforms the compared methods in terms of recognition accuracy rate and time consumption, thus holding significant practical value in an industrial production environment.

## 1. Introduction

**Figure 1:**
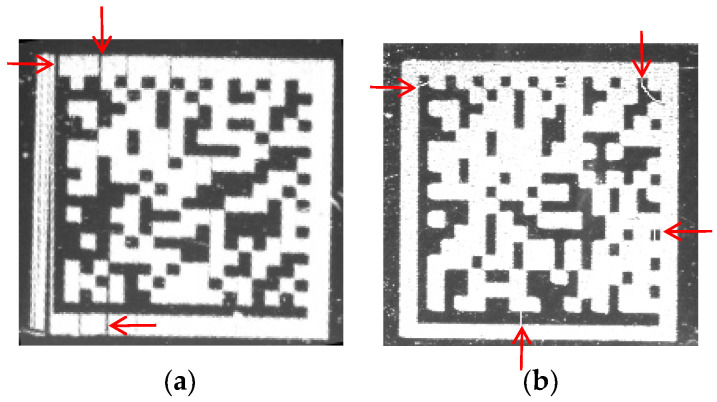
Interference issues marked by red arrows on the L-shaped solid edge (finder pattern) and dashed edge (timing pattern). (**a**) Protrusions. (**b**) Interruptions.

Data matrix (DM) codes are widely employed across various scenarios such as industrial automation [1,2], warehouse management [3,4], and smart logistics centers [5,6]. However, limitations in printing technology, insufficient precision in printing material processing, and scratches generated from contact between products often lead to the generation of low-quality DM code images in complex industrial production environments. These images are characterized by protrusions and interruptions on the L-shaped solid edge (finder pattern) and dashed edge (timing pattern), as shown in Figure 1. Such low-quality DM codes significantly diminish recognition rate. Therefore, it is urgent and meaningful to develop a method for recognizing low-quality DM codes with protrusions and interruptions on the L-shaped solid edge and dashed edge in complex industrial production environments.

In the case of limited protrusions and interruptions on the L-shaped solid edge, existing methods [7,8,9,10,11,12,13] based on L-shaped solid edge localization have achieved impressive results in DM code localization and recognition. However, when a certain degree of protrusions and interruptions occurs on the L-shaped solid edge, the success rate of these existing methods in terms of DM code localization sharply declines, which poses a significant challenge to DM code recognition. Nevertheless, the dashed edge possesses stronger regularity, robustness, and interference tolerance compared to the solid edge. Therefore, exploring ways to better utilize the characteristics of the dashed edge for DM code localization is a highly meaningful research direction.

It is not a trivial task to locate the L-shaped dashed edge reliably. Relying solely on simple and general properties, such as the adjacent black and white modules and the length ratio of black and white modules, may lead one to locate fake dashed edges. We observed that the center of the DM code provides valuable prior information that is of great assistance when locating the dashed edge. Consequently, it is worth exploring methods of finding the center of the DM code and appropriately leveraging the properties of the center for better locating the dashed edge.

**Figure 2:**
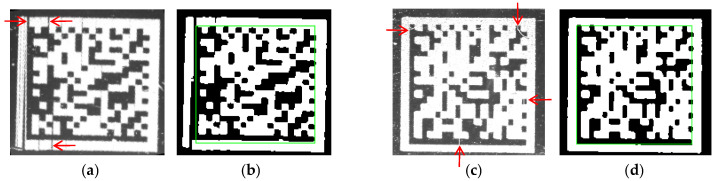
Rectangular region of the DM code without interference. (**a**) The DM code with protrusions marked by red arrows. (**b**) The DM code without protrusions in the green rectangle. (**c**) The DM code with interruptions marked by red arrows. (**d**) The DM code without interruptions in the green rectangle.

In order to address the interference issues caused by protrusions and interruptions on L-shaped solid and dashed edges, we in this paper propose a novel method for DM code recognition that utilizes the center of the DM code to locate the L-shaped dashed edge. Specifically, since the center of the DM code lacks distinct features, employing traditional image processing methods to acquire its position is exceedingly challenging. However, object detection methods [14,15,16,17,18,19,20,21,22,23,24,25,26,27,28,29,30,31,32,33] based on deep learning networks can locate the rectangular position of the DM code by identifying the features of the whole target, with the center of the rectangle approximating the center of the DM code. Therefore, we first employ YOLOv5 [34] to obtain the coarse position image of the DM code. Then, unlike existing methods [7,8,9,10,11,12,13] that rely on the L-shaped solid edge for locating DM code, we propose to locate DM code by first locating the L-shaped dashed edge using a two-level screening strategy. The strategy combines general constraints and central constraints deduced from the center of the coarse position of the DM code image. A precise position image of the DM code can be obtained from the rectangular region of the DM code which is determined by the L-shaped dashed edge. The green rectangle in Figure 2 illustrates the rectangular region of the DM code. In this region, protrusions on the L-shaped solid and dashed edges are excluded from the green rectangle in Figure 2b and interruptions are filled in Figure 2d. Finally, we utilize libdmtx [13] to recognize the DM code in the precise position image. Our proposed method effectively addresses the interference issues caused by protrusions and interruptions when locating the DM code, thereby improving the DM code recognition rate. Overall, our contributions in this paper can be summarized as follows:

1. In order to effectively address the interference issue posed by protrusions and interruptions on the L-shaped solid and dashed edges, we propose a novel localization framework consisted of coarse localization based on a deep learning network and fine localization based on the L-shaped dashed edge of the DM code.
2. To improve the accuracy of locating the L-shaped dashed edge, we propose a two-level screening strategy which combines general constraints and central constraints deduced from the center of the DM code.
3. Experimental results on various types of datasets demonstrate that the proposed method achieves a higher recognition accuracy rate and lower time consumption than the compared methods.

## 2. Related Work

DM code recognition mainly consists of two stages: localization and decoding. The localization stage determines the position of the DM code within an image, which is essential for decoding. Moreover, accurate and efficient localization can reduce the complexity and time consumption of decoding. Therefore, the majority of researchers [7,8,9,10,11,35,36] prioritize efforts to enhance the accuracy and efficiency of the localization to improve overall DM code recognition performance.

In early attempts, most methods focused on improving the performance of locating the DM code. Huang et al. [7] utilized line segment detection [37] to locate the “L” finder pattern of the DM code. Dai et al. [8] employed Hough transform to detect four vertices of DM code in order to locate the incomplete DM code on metal surfaces. Karrach et al. [9] proposed an efficient method which does not require extensive pre-processing for locating the DM code in production engineering. Pivarciova et al. [10] designed another efficient method mainly consisting locating the finder pattern and verification of the timing pattern to better locate the DM code. Although significant progress has been made with these methods, only consideration on ideal-quality DM code results in poor performance when they are applied in low-quality DM code environment. Therefore, utilizing deep learning-based methods with strong learning capability for localization is a new trend.

In recent years, several methods based on deep learning networks have been proposed for the detection and decoding of 2D barcode. For the detection of quick response (QR) code, several subsequent representative deep learning methods [20,38,39,40,41] showed impressive capabilities and gained increased attention. Chou et al. [42] employed a convolutional neural network to detect QR code with rotation and deformation. Hansen et al. [43] focused on developing a method for adapting the deep learning-based detector YOLO [20] to detect QR code in a real-time and reliable way. For the detection and decoding of DM code, Almeida et al. [35] proposed a new pipeline in which the detector is based on the deep learning Faster R-CNN [18] network and a conventional decoder method is used. However, the detection is time-consuming because of the large number of parameters in Faster R-CNN. To solve this issue, Almeida et al. [36] conducted a comprehensive analysis and compared experiments on representative deep learning-based object detection methods [18,19,21,25] with different backbones [39,40,41,44,45,46], and they proposed to use YOLOV4 [21] as the detector to acquire balanced detection performance between accuracy and latency. To deal with the problem that images captured by mobile cameras are usually of low quality with poor contrast, a deep learning-based method for industrial DM code was proposed by Liao et al. [47] to learn the colors of two adjacent modules of a DM symbol. An edge image was generated to reconstruct the final barcode image. While existing methods have made considerable progress, the lack of consideration for the the interference issues caused by protrusions and interruptions on L-shaped solid and dashed edges limits their effectiveness when applied in an industrial production environment.

For these interference issues, unlike existing methods [7,8,9,10,11,12,13] that rely on the L-shaped solid edge for DM code localization, our method relies on the L-shaped dashed edge with stronger interference-resisting capabilities for DM code localization. Existing methods [35,36] yield results that still contain the interference issues caused by protrusions and interruptions on the L-shaped solid and dashed edges when simply utilizing object detection methods based on deep learning network. In contrast, our approach leverages an object detection method to yield the center of the DM code, which is then used to build a two-level screening strategy combining general constraints and central constraints for locating the L-shaped dashed edge. This successfully eliminates the interference issues and achieves a higher recognition accuracy rate and lower time consumption than the compared methods.

## 3. Proposed Method

### 3.1. Overview

**Figure 3:**
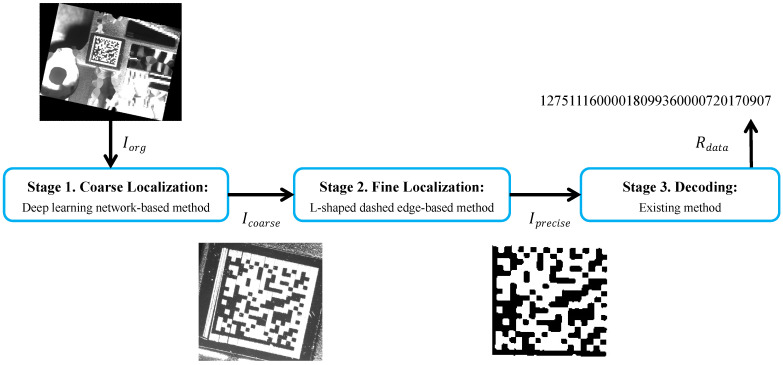
The overview architecture of our proposed method. This architecture consists of three processing stages: coarse localization, fine localization, and decoding.

We present the overview architecture of our proposed method in Figure 3. This architecture consists of three processing stages: coarse localization, fine localization, and decoding. In the first stage, we take the original DM code image $I_{org}$ as an input into coarse localization based on a deep learning network, yielding the coarse position image $I_{corase}$ of the DM code. In the second stage, $I_{corase}$ is passed on to a fine localization based on the L-shaped dashed edge, generating the precise position image $I_{precise}$ of the DM code. In the third stage, $I_{precise}$ is fed into an existing decoding method to output the data of the DM code $R_{data}$.

In what follows, we detail the three stages of our method, including the coarse localization stage (Section 3.2), the fine localization stage (Section 3.3), and the decoding stage (Section 3.4).

### 3.2. Coarse Localization

**Figure 4:**
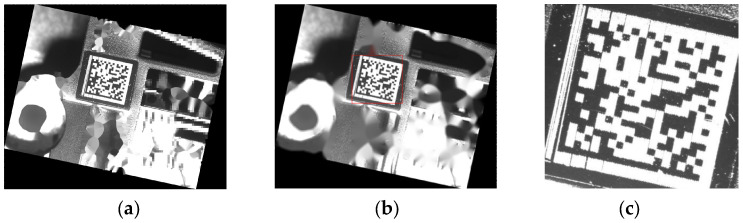
The input and output of coarse localization. (**a**) Input. (**b**) The coarse position of the DM code is in the red rectangle. (**c**) The coarse position image $I_{corase}$.

This processing stage aims to obtain the coarse position image $I_{corase}$ and the center of the DM code. In the first stage in Figure 3, we employ the YOLOv5 [34] method for coarse localization because YOLOv5 offers superior detection accuracy and latency compared to most object detection methods. The original DM code image used as an input, as shown in Figure 4a, is passed to YOLOv5. The red rectangle in Figure 4b is the result detected by YOLOv5. Thanks to the powerful learning capabilities of deep learning methods, even when the solid and dashed edges of the DM code are affected by protrusions and interruptions, the detected results still contain the entire DM code instead of an incomplete DM code. The outputs of this stage are the coarse position image $I_{corase}$, as shown in Figure 4c, and the center of the DM code, which is the center of $I_{corase}$. Importantly, for low-resolution DM codes in high-resolution images, low-resolution DM code images evidently reduce the computational resources required for the two subsequent processing stages. Meanwhile, low-resolution DM code images also reduce the complexity of dashed edges localization and the time consumption of DM code recognition.

It is noteworthy that existing DM code recognition methods struggle to effectively locate and recognize DM code with protrusions and interruptions on solid and dashed edges. However, our proposed method can handle it well. Therefore, the next fine localization stage details how to exclude protrusions and interruptions on the dashed edge.

### 3.3. Fine Localization

**Figure 5:**
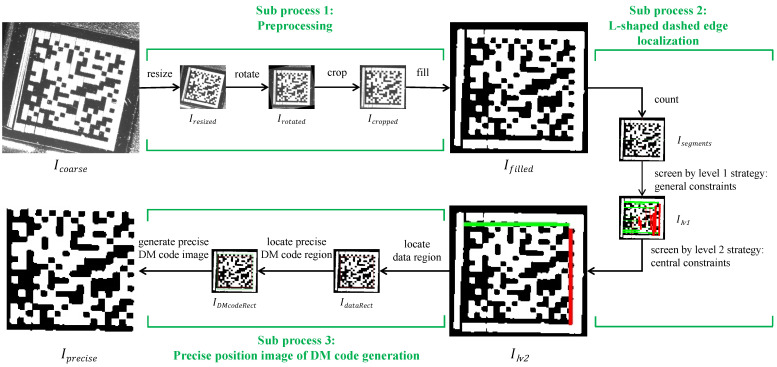
The procedure overview diagram of fine localization based on the L-shaped dashed edge.

The main target of this processing stage is to obtain the precise position image $I_{precise}$ of the DM code without protrusions and interruptions on the L-shaped solid and dashed edges. We present the procedure overview diagram of fine localization based on the L-shaped dashed edge in Figure 5. This procedure consists of three subprocesses: preprocessing, L-shaped dashed edge localization, and generation of the DM code’s precise position image.

#### 3.3.1. Prepocessing

The objective of the first subprocess is to obtain the upright DM code image without interruptions on the L-shaped solid and dashed edges. As shown in Figure 5, an upright DM code image $I_{\mathrm{filled}}$ means that the solid and dashed edges of the DM code are parallel or perpendicular to the x-axis of the image. This subprocess consists of four steps: resizing, rotating, cropping, and filling.

(1) Resizing. To facilitate subsequent processing, we resize the coarse position image $I_{corase}$ to obtain the resized image $I_{\mathrm{resized}}$ which is calculated as follows:

(1) $$\left(\begin{array}{c} x_{rs}\\ y_{rs}\\ 1 \end{array}\right)=\left(\begin{array}{ccc} r & 0 & 0\\ 0 & r & 0\\ 0 & 0 & 1 \end{array}\right)*\left(\begin{array}{c} x_{cs}\\ y_{cs}\\ 1 \end{array}\right)$$

where $\left(x_{rs},y_{rs}\right)$ is the pixel coordinate of image $I_{\mathrm{resized}}$, and $\left(x_{cs},y_{cs}\right)$ is the pixel coordinates of image $I_{\mathrm{corase}}$. *r* is a scale factor which is calculated as follows:

(2) $$r=max\left(H_{cs},W_{cs}\right)/l_{rs}$$

where $H_{cs}$ and $W_{cs}$ are the height and width of image $I_{corase}$. $l_{rs}$ is the resized length of the long side of image $I_{resized}$, and we empirically set $l_{rs}$ to 200. Function max selects the max value of all elements. The height $H_{rs}$ and width $W_{rs}$ of the resized image $I_{resized}$ are calculated as follows:

(3) $$\begin{array}{c} H_{rs}=H_{cs}*r\\ W_{rs}=W_{cs}*r \end{array}$$

(2) Rotating. To reduce the complexity of L-shaped dashed edge localization, we rotate the resized image $I_{resized}$ until it is an upright DM code image $I_{rotated}$ with interruptions by three substeps. First, we employ the Canny method [48] to extract the edge image $I_{edge}$ from $I_{resized}$. Then, we employ the Hough method [49] to identify white lines in the edge image, and each line must have a point count that exceeds a specified threshold value. Here, we set this threshold value to 35. The most frequent angle among all lines is determined to be the rotation angle θ. Finally, the image $I_{rotated}$ can be expressed as follows:

(4) $$\left(\begin{array}{c} x_{rt}\\ y_{rt}\\ 1 \end{array}\right)=\left(\begin{array}{ccc} cos\theta & sin\theta & 0\\ -sin\theta & cos\theta & 0\\ 0 & 0 & 1 \end{array}\right)*\left(\begin{array}{c} x_{rs}-x_{c}\\ y_{rs}-y_{c}\\ 1 \end{array}\right)+\left(\begin{array}{c} x_{c}\\ y_{c}\\ 0 \end{array}\right)$$

where $\left(x_{rt},y_{rt}\right)$ is the pixel coordinate of image $I_{rotated}$. $\left(x_{rs},y_{rs}\right)$ is the pixel coordinate of image $I_{resized}$. $\left(x_{c},y_{c}\right)$ is the rotated center coordinate of image $I_{resized}$. The width and height of $I_{rotated}$ are the same as $I_{resized}$.

We found that the occupied width of the DM code in the image is shorter after rotation than before, which leads to the formation of invalid border regions. It is beneficial to improving the effect of subsequent central constraints on locating the L-shaped dashed edge and avoiding the interference of a fake dashed edge if these invalid border regions are eliminated. Therefore, we crop these regions of the rotated image $I_{rotated}$ in next processing step.

**Figure 6:**
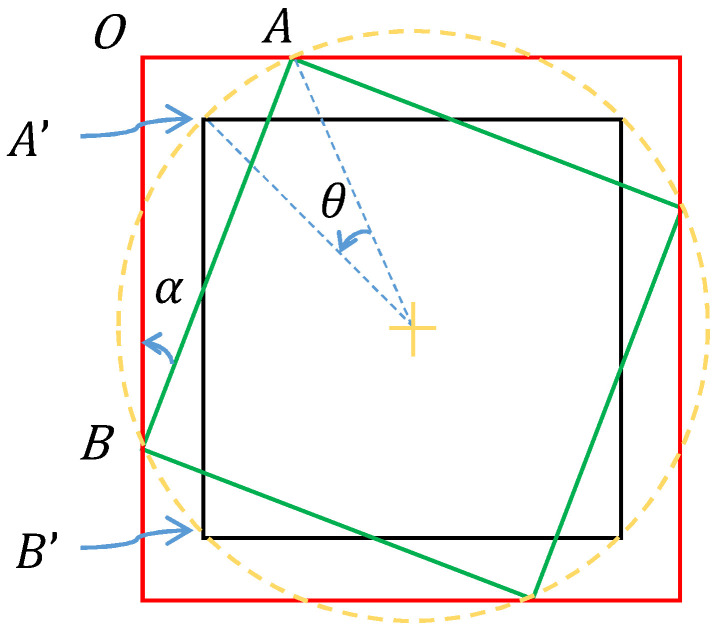
The schematic diagram used to illustrate the cropping of invalid border regions.

(3) Cropping. We crop the width *d* of invalid border regions in rotated image $I_{rotated}$ in order to obtain the cropped image $I_{cropped}$. We assume that all four vertices of the DM code before rotation are located on the boundary of the image, that the center of the image is the center of the DM code, and that the shape of the image is square. Thus, we can draw a schematic diagram as shown in Figure 6. The green square represents the DM code whose four vertices are located on the red square that stands for the image. The yellow cross represents the center of the DM code and image. The yellow circle indicates the vertex trajectory formed by rotating the DM code 360 degree. Further, we assume that the black square represents the rotated DM code after the counterclockwise rotation angle θ of the green DM code. Therefore, the region between the black square and the red square is the invalid border region we need to crop. Since the vertex *A* becomes vertex $A^{\prime }$ after rotation, the height difference between vertex *A* and vertex $A^{\prime }$ is our desired cropped width *d* of the invalid border regions.

In the coordinate system of the image, we assume that $\left(x_{A},y_{A}\right)$ is the pixel coordinate of *A* and that $\left(x_{A^{\prime }},y_{A^{\prime }}\right)$ is the pixel coordinate of A’. Since point *A* lies on the x-axis, namely $y_{A}$ equals 0, *d* is equal to the value of $y_{A^{\prime }}$. Given that the point *A* becomes point *A*’ after rotating angle θ, $y_{A^{\prime }}$ can be expressed by Equation (4) as follows:

(5) $$y_{A^{\prime }}=-\left(x_{A}-x_{c}\right)sin\theta -y_{c}cos\theta +y_{c}$$

where $\left(x_{c},y_{c}\right)$ represents the center coordinates of the image. Therefore, $y_{A^{\prime }}$ can be calculated from the above equation if $x_{A}$ is known.As shown in Figure 6, we assume that α represents the angel ABO in right triangle ABO. The tangent of angle α can be expressed as follows:

(6) $$tan\alpha =\frac{AO}{BO}=\frac{x_{A}}{W-x_{A}}.$$

After transformation, $x_{A}$ can be expressed as follows:

(7) $$x_{A}=\frac{Wtan\alpha }{1+tan\alpha }$$

where *W* represents the width of the image before rotation. Angle α rotated from side AB to A’B’ is equal to angle θ rotated from point *A* to point A’. Finally, $y_{A^{\prime }}$ can be expressed by combing Equations (5) and (7) as follows:

(8) $$y_{A^{\prime }}=W\left[1-\frac{cos\theta }{2}-\left(\frac{tan\theta }{1+tan\theta }-\frac{1}{2}\right)sin\theta \right]$$

(4) Filling. To remove the interruption on L-shaped solid and dashed edges of DM code in image $I_{cropped}$, we first utilize the Ostu method [50] to generate the binary image, then fill the interruptions of binary image by utilizing morphological erode and dilate operations to obtain the interruption filled image $I_{filled}$ as shown in Figure 5. The structure element of morphological operations is 3 × 3 matrix.

#### 3.3.2. L-Shaped Dashed Edge Localization

The target of the second subprocess is to locate the L-shaped dashed edge as marked in $I_{lv2}$ of Figure 5. This subprocess involves two steps: counting and screening.

(1) Counting. We count the continuous black and white pixel segments of all rows and columns in image $I_{filled}$ according to the horizontal and vertical directions. The single row and column of all segments in the horizontal and vertical directions are denoted as $S_{h}\left(r\right)$ and $S_{v}\left(c\right)$, respectively, where r∈[0,H), c∈[0,W). *H* and *W* are the height and width of the image.

(2) Screening. To locate all rows and columns of the L-shaped dashed edge, we specially design a two-level screening strategy which combines the general constraints and central constraints by fully utilizing the central properties of the DM code. The single row and column of dashed edges in the horizontal and vertical directions are denoted as $D_{h}\left(r\right)$ and $D_{v}\left(c\right)$, respectively. The details of the two-level screening strategy are as follows:

Level 1: For the horizontal direction, $s_{h}\left(r,j\right)$ represents the *j*-th segment of the *r*-th row $S_{h}\left(r\right)$, where r∈[0,H), $j\in [0,N_{h}\left(r\right))$, and $N_{h}\left(r\right)$ refer to the number of segments in the *r*-th row. $c_{h}\left(r,j\right)$ represents the color of $s_{h}\left(r,j\right)$, and the values 0 and 1 stand for the colors black and white, respectively. $l_{h}\left(r,j\right)$ denotes the length of $s_{h}\left(r,j\right)$. $i_{st}$ and $i_{ed}$ represent the index in $S_{h}\left(r\right)$ of the starting segment and ending segment of the dashed edge, respectively. The specific row of the dashed edge in the horizontal direction $D_{h}\left(r\right)$ should satisfy the following general constraints:

1. The color of the starting segment is black, and the starting segment is not the first segment. The equation can be expressed as $c_{h}\left(r,i_{st}\right)=0$, where $i_{st}\in \left[1,N_{h}\left(r\right)-1\right]$.
2. The length ratio between the starting segment and each subsequent segment should fall within the range $\left[r_{min},r_{max}\right]$. Here, we set 0.5 and 2 to $r_{min}$ and $r_{max}$, respectively. The equation can be expressed as $r_{min}\leq l_{h}\left(r,i_{st}\right)/l_{h}\left(r,j\right)\leq r_{max}$, where $j\in [i_{st}+1,N_{h}\left(r\right)-1]$.
3. The color of the ending segment is black, and the ending segment is not the last segment. There is at least one segment between the starting segment and the ending segment. The equation can be expressed as $c_{h}\left(r,i_{ed}\right)=0$, where $i_{ed}\in \left[i_{st}+2,N_{h}\left(r\right)-1\right)$.

For the vertical direction, $D_{v}\left(c\right)$ should satisfy the same constraints as $D_{h}\left(r\right)$.

Level 2: For the horizontal direction, $h_{h}\left(r,j\right)$ and $t_{h}\left(r,j\right)$ represent the head and tail column index of $s_{h}\left(r,j\right)$, respectively. $n_{h,l}\left(r\right)$ and $n_{h,r}\left(r\right)$ stand for the number of segments located to the left and right of the vertical middle line of the image. $y_{h}\left(r,j\right)$ represents the row value of $s_{h}\left(r,j\right)$. $D_{h}\left(r\right)$ should satisfy the following central constraints:

1. The starting segment is located to the left of the vertical middle line of the image. The equation can be expressed as $t_{h}\left(r,i_{st}\right)<W/2$.
2. The ending segment is located to the right of the vertical middle line of the image. The equation can be expressed as $h_{h}\left(r,i_{ed}\right)>W/2$.
3. The number of segments located to the left and right of the vertical middle line of the image is greater than or equal to 4. The equation can be expressed as $n_{h,l}\left(r\right)\geq 4,n_{h,r}\left(r\right)\geq 4$.
4. The difference in the number of segments between the left and right side of the vertical middle line of the image is less than or equal to 2. The equation can be expressed as $|n_{h,l}\left(r\right)-n_{h,r}\left(r\right)|\leq 2$.
5. The distance between the horizontal dashed edge and the horizontal middle line of the image is greater than one third of the image’s height. The equation can be expressed as $|y_{h}\left(r,j\right)-H/2|>H/3$.

For the vertical direction, $D_{v}\left(c\right)$ should satisfy the same constraints as $D_{h}\left(r\right)$.

**Figure 7:**
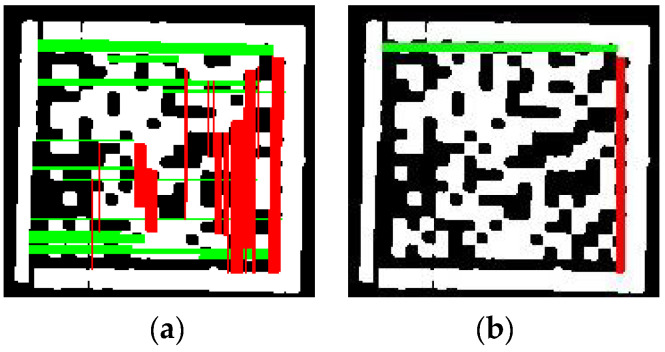
The results of two-level screening strategy. (**a**) Level 1: general constraints. The red lines represent the horizontal dashed edges screened by Level 1. The green lines represent the vertical dashed edges screened by Level 1. (**b**) Level 1 + 2: general and central constraints. The red lines represent the horizontal dashed edges screened by Level 1 + 2. The green lines represent the vertical dashed edges screened by Level 1 + 2.

As shown in Figure 7a, only employing the general constraints is insufficient to eliminate fake dashed edges located in either the left or right half of the image. However, these fake edges can be eliminated as shown in Figure 7b by combining the general constraints and the central constraints, which demonstrates that our two-level screening strategy is beneficial for locating the correct dashed edges of the DM code.

#### 3.3.3. Generation of the Precise Position Image of the DM Code

The third subprocess aims to generate the precise position image of the DM code with the protrusions on L-shaped solid and dashed edges removed. As shown in Figure 5, this subprocess consists of three steps: localization of the data region of the DM code, localization of the precise position region of the DM code, and generation of the precise position image of the DM code.

**Figure 8:**
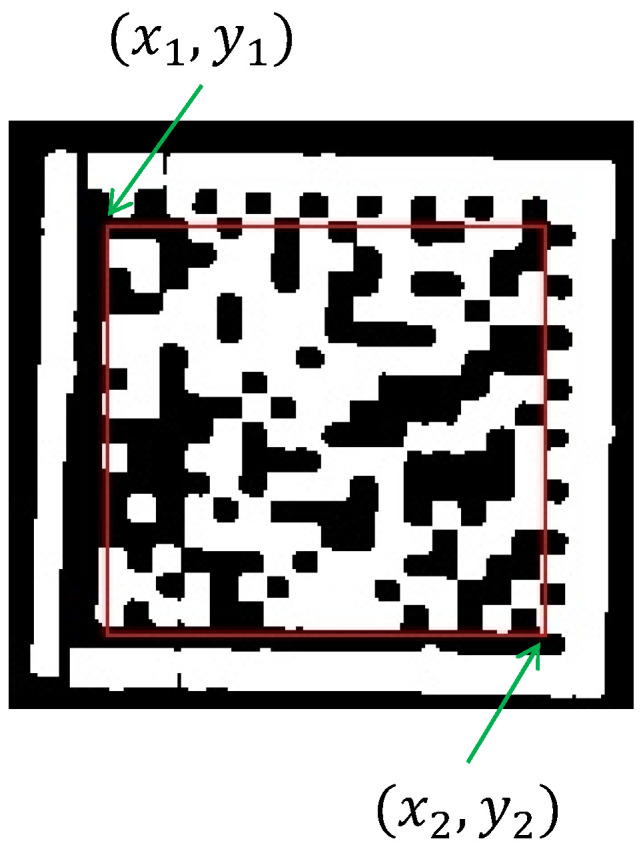
The top-left coordinate $\left(x_{1},y_{1}\right)$ and bottom-right coordinate $\left(x_{2},y_{2}\right)$ of data region $r_{data}$ marked by red rectangle.

**Figure 9:**
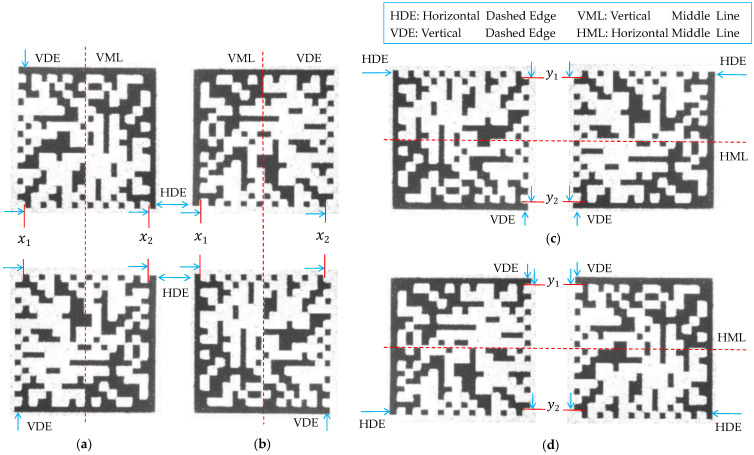
Cases for determining $x_{1}$, $x_{2}$, $y_{1}$, and $y_{2}$ of the data region $r_{data}$. (**a**) Determining $x_{1}$, $x_{2}$ when the VDE is located to the left of the VML of the image. (**b**) Determining $x_{1}$, $x_{2}$ when the VDE is located to the right of the VML of the image. (**c**) Determining $y_{1}$, $y_{2}$ when the HDE is located above the HML of the image. (**d**) Determining $y_{1}$, $y_{2}$ when the HDE is located below the HML of the image.

**Figure 10:**
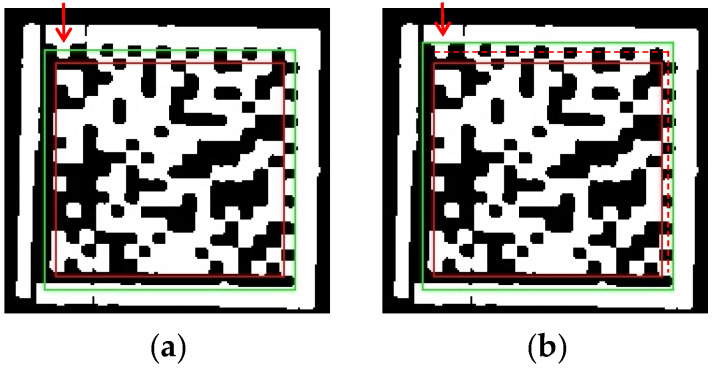
The results from comparing the two expansion methods for dashed edges. The red and green rectangles represent $r_{data}$ and $r_{pp}$, respectively. The two red arrows point out the differences between the two results. The horizontal and vertical red dashed lines represent the middle row and column of the dashed edges, respectively. (**a**) The results of the expansion method based on the sides of $r_{data}$ near the two dashed edges. (**b**) The results of the expansion method based on the middle row or column of the dashed edges.

**Figure 11:**
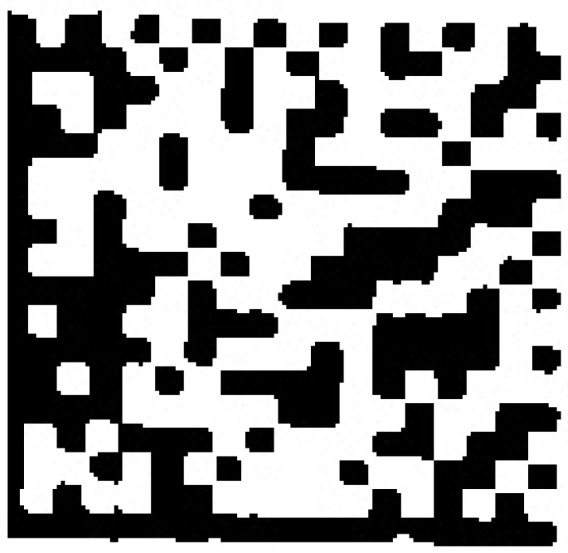
The precision position image $I_{precise}$ of the DM code.

(1) Localization of the data region of the DM code. To determine the data region $r_{data}$ of the DM code, we design special rules for the horizontal dashed edge and vertical dashed edge located in the second subprocess. We first select the middle row $D_{h}\left(M_{h}\right)$ and middle column $D_{v}\left(M_{v}\right)$ of the horizontal dashed edge and vertical dashed edge, with $M_{h}$ and $M_{v}$ being the row and column index of the image, respectively. Then, we select two horizontal coordinates, $x_{1}$ and $x_{2}$, on $D_{h}\left(M_{h}\right)$ and two vertical coordinates, $y_{1}$ and $y_{2}$, on $D_{v}\left(M_{v}\right)$. Finally, points $\left(x_{1},y_{1}\right)$ and $\left(x_{2},y_{2}\right)$ are used as the top-left and bottom-right coordinates of the rectangle, respectively, to form the data region $r_{data}$ of the DM code, as shown in the red box in Figure 8. In detail, $x_{1}$, $x_{2}$, $y_{1}$, and $y_{2}$ can be obtained as follows:

1. As shown in Figure 9a, when $D_{v}\left(M_{v}\right)$ is located to the left of the vertical middle line of the image, i.e., $M_{v}<W/2$, $x_{1}$ and $x_{2}$ are assigned as the head index of the starting segment and head index of the ending segment of $D_{h}\left(M_{h}\right)$, respectively. The equation can be expressed as $\left\{\begin{array}{c} x_{1}=h_{h}\left(M_{h},i_{st}\right)\\ x_{2}=h_{h}\left(M_{h},i_{ed}\right) \end{array}\right.$.
2. As shown in Figure 9b, when $D_{v}\left(M_{v}\right)$ is located to the right of the vertical middle line of the image, i.e., $W/2<M_{v}$, $x_{1}$ and $x_{2}$ are assigned as the tail index of the starting segment and tail index of the ending segment of $D_{h}\left(M_{h}\right)$, respectively. The equation can be expressed as $\left\{\begin{array}{c} x_{1}=t_{h}\left(M_{h},i_{st}\right)\\ x_{2}=t_{h}\left(M_{h},i_{ed}\right) \end{array}\right.$.
3. As shown in Figure 9c, when $D_{h}\left(M_{h}\right)$ is located above the horizontal middle line of the image, i.e., $M_{h}<H/2$, $y_{1}$ and $y_{2}$ are assigned as the head index of the starting segment and head index of the ending segment of $D_{v}\left(M_{v}\right)$, respectively. The equation can be expressed as $\left\{\begin{array}{c} y_{1}=h_{v}\left(M_{v},i_{st}\right)\\ y_{2}=h_{v}\left(M_{v},i_{ed}\right) \end{array}\right.$.
4. As shown in Figure 9d, when $D_{h}\left(M_{h}\right)$ is located below the horizontal middle line of the image, i.e., $H/2<M_{h}$, $y_{1}$ and $y_{2}$ are assigned as the tail index of the starting segment and tail index of the ending segment of $D_{v}\left(M_{v}\right)$, respectively. The equation can be expressed as $\left\{\begin{array}{c} y_{1}=t_{v}\left(M_{v},i_{st}\right)\\ y_{2}=t_{v}\left(M_{v},i_{ed}\right) \end{array}\right.$.

(2) Localization of the precise position region of the DM code. To determine the precise position region $r_{pp}$ of the DM code, we expand the data region $r_{data}$ of the DM code along four directions of the rectangular edge. According to our observations, the dashed edges of the DM code are farther from the sides of the data region than the solid edges are in industrial production environments. Directly expanding the same width for solid and dashed edges may cause the loss of crucial information from the dashed edges, leading to the failure of DM code recognition. Therefore, we use different methods for expanding the solid and dashed edges to better adapt to the low-quality DM code images found in industrial production environments. Specifically, we assume that ${\bar{l}}_{h}$ and ${\bar{l}}_{v}$ represent the average length of black segments along a horizontal dashed edge and a vertical dashed edge, respectively. For horizontal and vertical solid edges, we expand the width ${\bar{l}}_{h}$ and ${\bar{l}}_{v}$ based on the two sides of $r_{data}$ near the two solid edges. For horizontal and vertical dashed edges, we expand the width ${\bar{l}}_{h}$/2 and ${\bar{l}}_{v}$/2 based on the $D_{h}\left(M_{h}\right)$ and $D_{v}\left(M_{v}\right)$ instead of two sides of $r_{data}$ near the two dashed edges. As shown in Figure 10, the results from comparing the two expansion methods for dashed edges indicate that the method based on the middle row or column of the dashed edge in Figure 10b can retain more crucial dashed edge information than the method based on the sides of $r_{data}$ near the two dashed edges in Figure 10a. The green rectangle in Figure 10b is our desired precise position region $r_{pp}$ of the DM code.

(3) Generation of the precise position image of the DM code. We first extract the image of rectangle region $r_{pp}$ from image $I_{filled}$. Then, we add a white border with 5 pixels into the extracted image to generate the precision position image $I_{precise}$ of the DM code. As shown in Figure 11, the protrusions on the L-shaped solid and dashed edges in $I_{precise}$ have been removed.

### 3.4. Decoding

This processing stage is aimed at obtaining the data of the DM code $R_{data}$. Through the preceding two processing stages, a precise position image of the DM code $I_{precise}$ without any protrusions or interruptions on the L-shaped solid and dashed edges has been generated, as shown in Figure 11. Of the current DM code decoding methods, we selected the libdmtx method, which offers optimal performance and speed, to obtain the data of the DM code $R_{data}$.

## 4. Experimental Results and Analyses

In this section, we primarily describe the experimental setup for comparison, present the comparative results, and conduct detailed analysis. First, we describe the implementation details and experiment settings including the test dataset, comparison methods, and evaluation metrics. Subsequently, to demonstrate the effectiveness and superiority of our proposed method, we present and analyze the comparison results of recognition accuracy and time consumption between our proposed method and the compared methods. Finally, to further explain the details of the proposed method, we present time consumption analysis and conduct a series of ablation studies for the proposed method.

### 4.1. Implementation Details

We implemented the proposed method with the OpenCV(C++) library on a PC with a 3.2 GHz Intel CPU and an Nvidia 2060s GPU. We selected the YOLOv5s-v7.0 for coarse localization. In order to improve the detection accuracy of YOLOv5 [34] for the DM code given the interference of protrusions and interruptions, we added 204 images with and without interference to the training dataset.

### 4.2. Experiment Settings

**Figure 12:**
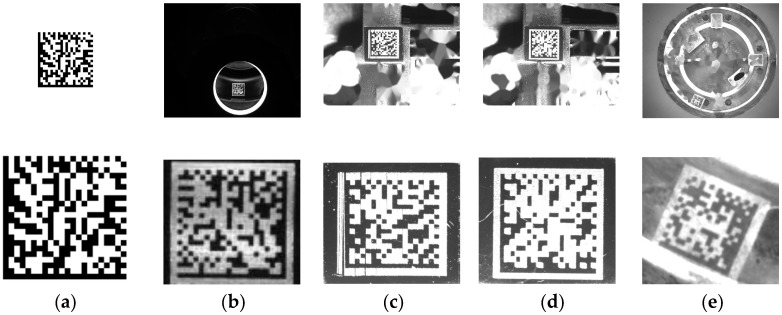
Example images of the five types. The first row presents the original DM code images, and the second row presents zoomed-in images of the DM codes. (**a**) Type-1. (**b**) Type-2. (**c**) Type-3. (**d**) Type-4. (**e**) Type-5.

Test dataset: To test the effectiveness and general capacity of the proposed method, we selected a test dataset which includes five types of images. These five types consist of perfect DM codes without interference, real DM codes without interference, DM codes with protrusions, DM codes with interruptions, and rotated DM codes. We denoted the five types as Type-1, Type-2, Type-3, Type-4, and Type-5, respectively. The test dataset contains a total of 265 images, and the number of each type is shown in Table 1. We present the several sample image of each type in Figure 12.

Compared methods: The mainstream methods of DM recognition are zxing [12] and libdmtx [13]. Unlike our proposed method recognize the DM code based on the L-shaped dashed edge localization, these two methods recognize the DM code based on the L-shaped solid edge localization. Therefore, we compared our method with these two methods which were implemented with python framework zxing-cpp 1.4.0 and pylibdmtx 0.1.10 respectively. Additionally, we compared our method with two commercial software (onbarcode [51] trial version and inlite [52] 12.0.7675).

Evaluation metrics: Obviously, we need to recognize the DM code on products with high accuracy and low time consumption in industrial production environments. Consequently, we selected these two metrics to measure the performance of our method against the compared methods.

### 4.3. Recognition Accuracy Rate Comparison

**Figure 13:**
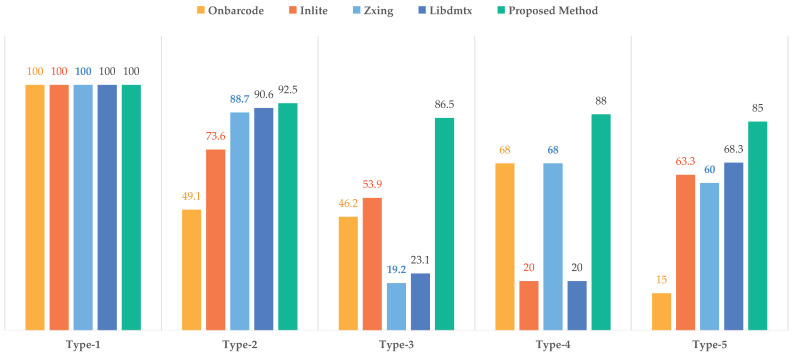
The recognition accuracy rate (%) of five methods.

The compared results of recognition accuracy rate are shown in Figure 13. As can be seen from the comparison results of each type, our method exhibits superior adaptability and achieves the highest recognition accuracy rate, which is credited to the design of the dashed edge localization in our proposed method. The specific analysis results are as follows:

1. Since the DM code exhibits no interference or module distortion in Type-1, all five methods can recognize all images correctly.
2. Although the DM code has no interference in Type-2, there are two differences compared to Type-1: (1) The modules on the edge of the DM code are irregular and the sizes of modules are inconsistent; (2) There are gaps between rows of different DM code modules. These two differences pose some challenges for recognition methods. According to the compared results, our proposed method achieved a better recognition accuracy rate than onbarcode, inlite, zxing, and libdmtx.
3. Our proposed method achieved a significantly higher recognition accuracy rate compared to onbarcode, inlite, zxing, and libdmtx for Type-3. Zxing and libdmtx achieved relative low recognition rates because they lack enough consideration for the interference of protrusion. Onbarcode and inlite achieved recognition rate of about 50%. However, even in the presence of protrusion interference on solid and dashed edges of DM codes, the proposed method can still recognize them correctly with high success rates. This success is attributed to the central constraints employed in our proposed method for the precise localization of dashed edges.
4. Our proposed method can recognize the most DM codes with interruption interference in Type-4. This is because we conduct dilate and erode operations to fill the instances of interruption interference on L-shaped solid and dashed edges. Onbarcode and zxing can recognize more than half of the codes, demonstrating their ability to resist interruption interference. However, inlite and libdmtx fail to recognize most of them, indicating little consideration for interruption interference.
5. For rotated DM code images in Type-5, our proposed method achieved a recognition accuracy of 85%. This indicates that our method not only possesses resistance to protrusion and interruption interference but also that it exhibits resistance to 2D rotation. This expands the application scenarios of our proposed method into real production environments.

### 4.4. Time Consumption Comparison

Taking into account the background and quality of the DM code within the image, we classified all of the test images into two groups to compare time consumption. Group-1 consists of images (Type-1) with clean backgrounds and perfect DM codes generated by onbarcode and inlite. Group-2 (Type-2∼5) comprises real-world images with impure backgrounds and interference in the DM code. Table 2 presents the comparison results for time consumption on all recognized DM codes from the two groups. In the results of Group-1, in terms of average time consumption, libdmtx was the fastest, followed by zxing and the proposed method. In terms of stability, all five methods exhibited relatively stable performance. This is because the compared methods can quickly eliminate clean backgrounds and locate the DM code. In the results of Group-2, in terms of average time consumption, the proposed method was the fastest, followed by zxing, onbarcode, inlite, and libdmtx. In terms of stability, the four compared methods had larger standard deviation than our proposed method. This is because there are some similar localization features of the DM code in the background, which results in the compared methods wasting more time in locating and recognizing in these areas. However, in our proposed method, the deep learning method fully utilized GPU for rapid coarse localization, significantly reducing the time consumption of subsequent processing stages.

### 4.5. Time Consumption Analysis

**Figure 14:**
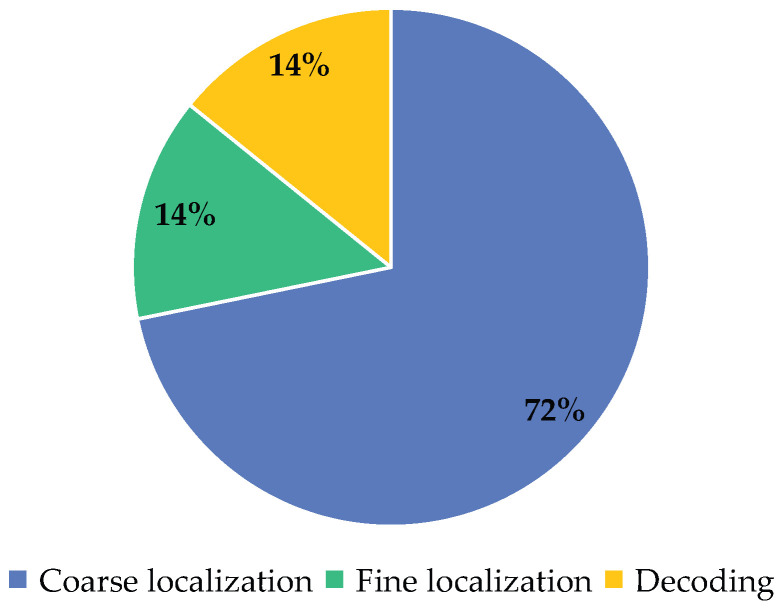
The proportion of time consumption for the three stages of the proposed method.

Figure 14 shows the proportion of time consumption for the three stages of the proposed method on all recognized DM codes. Obviously, the coarse localization based on the YOLOv5 method accounts for the largest proportion of time consumption, reaching 72%, while the fine localization and decoding based on traditional image processing methods each account for 14%. This indicates that the majority of time consumed in our proposed method is spent on the coarse localization stage. The output results of coarse localization reduce a significant amount of ineffective processing regions, thus saving time for fine localization and decoding. Compared to the time-consuming operation of directly using the libdmtx method to traverse the entire image searching for the L-shaped solid edge, our proposed method makes reasonable use of the libdmtx method, resulting in a significant reduction in time consumption.

### 4.6. Ablation Study

We conducted ablation studies to confirm the impact of core processing modules in our proposed method for recognizing DM codes. Four cases are described as follows: (1) Case 1: Complete method; (2) Case 2: Excluding the YOLOv5 method for coarse localization; (3) Case 3: Excluding the module for cropping borders after rotation in the preprocessing subprocess in the second stage; (4) Case 4: Excluding central constraints in the L-shaped dashed edge localization subprocess in the second stage.

Based on the results shown in Table 3, we can draw the following conclusions:

1. The complete method achieves the highest recognition accuracy rate, whereas the recognition accuracy rates for the other three cases diminish, with some even failing to recognize all images. This demonstrates the effectiveness of the core modules in our proposed method.
2. The processing modules for coarse localization and central constraints are essential for the recognition of DM codes. Without these modules, recognition of all DM code images is not feasible.
3. Cropping borders has a certain impact on the recognition accuracy rate. In the case of rotated DM codes, the requirement of the fifth point in Level 2 of the central constraints cannot be satisfied if the invalid borders are not cropped. This will result in the inability to locate dashed edges. However, in the case of upright DM codes, no invalid borders need to be cropped. Therefore, excluding the module for cropping borders has no impact on the localization of dashed edges and the recognition of DM codes.

## 5. Conclusions

In this paper, we proposed a novel DM code recognition method based on locating the L-shaped dashed edge by combining the center of the DM code. The proposed method can recognize low-quality images of DM codes with protrusions and interruptions on the L-shaped solid and dashed edges with high accuracy and low time consumption. Based on the experimental results presented in this paper, we can draw the following conclusions: (1) The L-shaped dashed edge can provide stronger regularity, robustness, and interference tolerance for DM code localization than the L-shaped solid edge; (2) The center of the DM code can provide more useful constraints for L-shaped dashed edge localization than general constraints; (3) The image with less content and lower resolution generated from coarse localization can reduce the complexity and time consumption of subsequent processing stages; (4) For DM codes with protrusions and interruptions on the L-shaped solid and dashed edges, our proposed method is a better solution than the compared methods in terms of recognition accuracy rate and time consumption. Therefore, it holds significant application value in real-world industrial production environments.

## Figures and Tables

**Table 1:** The number of images showing each of the five types.

Type	Image Number
1	50
2	53
3	52
4	50
5	60

**Table 2:** The time consumption (ms) of the five methods. Only the recognized DM code images are counted. The best result is in bold.

Group	Onbarcode		Inlite		Zxing		Libdmtx		Proposed Method	
	Mean	StdDev	Mean	StdDev	Mean	StdDev	Mean	StdDev	Mean	StdDev
1	122	5	57	8	8	**2**	**5**	**2**	19	5
2	150	78	229	331	22	35	427	2242	**20**	**6**

**Table 3:** The results of ablation studies for core processing modules. ✓ means the case includes this module and ✗ means the case excludes this module.

Case	Coarse Localization	Cropping Border	Central Constraints	Recognition Accuracy Rate
1	✓	✓	✓	90.2%
2	✗	✓	✓	0%
3	✓	✗	✓	75.5%
4	✓	✓	✗	0%

## Data Availability

The data cannot be made publicly available because the image contains the details of product which represents the highest level of confidential information within the company.
